# Monitoring of Gait Parameters in Post-Stroke Individuals: A Feasibility Study Using RGB-D Sensors

**DOI:** 10.3390/s21175945

**Published:** 2021-09-04

**Authors:** Claudia Ferraris, Veronica Cimolin, Luca Vismara, Valerio Votta, Gianluca Amprimo, Riccardo Cremascoli, Manuela Galli, Roberto Nerino, Alessandro Mauro, Lorenzo Priano

**Affiliations:** 1 Institute of Electronics, Computer and Telecommunication Engineering, National Research Council, Corso Duca degli Abruzzi 24, 10129 Torino, Italy; valerio.votta@ieiit.cnr.it (V.V.); gianluca.amprimo@ieiit.cnr.it (G.A.); roberto.nerino@ieiit.cnr.it (R.N.); 2 Department of Electronics, Information and Bioengineering, Politecnico di Milano, Piazza Leonardo da Vinci 32, 20133 Milano, Italy; veronica.cimolin@polimi.it (V.C.); manuela.galli@polimi.it (M.G.); 3 Istituto Auxologico Italiano, IRCCS, Department of Neurology and Neurorehabilitation, S. Giuseppe Hospital, Oggebbio (Piancavallo), 28824 Verbania, Italy; lucavisma@hotmail.com (L.V.); riccardo.cremascoli@unito.it (R.C.); alessandro.mauro@unito.it (A.M.); lorenzo.priano@unito.it (L.P.); 4 Department of Neurosciences, University of Turin, Via Cherasco 15, 10100 Torino, Italy

**Keywords:** RGB-D sensors, gait analysis, stroke, automated assessment, remote monitoring, rehabilitation, ecological setting

## Abstract

Stroke is one of the most significant causes of permanent functional impairment and severe motor disability. Hemiplegia or hemiparesis are common consequences of the acute event, which negatively impacts daily life and requires continuous rehabilitation treatments to favor partial or complete recovery and, consequently, to regain autonomy, independence, and safety in daily activities. Gait impairments are frequent in stroke survivors. The accurate assessment of gait anomalies is therefore crucial and a major focus of neurorehabilitation programs to prevent falls or injuries. This study aims to estimate, using a single RGB-D sensor, gait patterns and parameters on a short walkway. This solution may be suitable for monitoring the improvement or worsening of gait disorders, including in domestic and unsupervised scenarios. For this purpose, some of the most relevant spatiotemporal parameters, estimated by the proposed solution on a cohort of post-stroke individuals, were compared with those estimated by a gold standard system for a simultaneous instrumented 3D gait analysis. Preliminary results indicate good agreement, accuracy, and correlation between the gait parameters estimated by the two systems. This suggests that the proposed solution may be employed as an intermediate tool for gait analysis in environments where gold standard systems are impractical, such as home and ecological settings in real-life contexts.

## 1. Introduction

Stroke and cerebrovascular diseases are prominent causes of disability: The consequences of the acute event negatively impact the quality of life and require long-term rehabilitation [1,2] to favor the partial or total recovery of impaired functions. Stroke severely affects several motor skills at different levels as a result of muscular weakness or partial paralysis on one side of the body. In particular, common disabilities concern the motor functions of upper and lower limbs, present in over 80% of stroke survival cases [3]. Moreover, visual impairments or peripheral field loss, mostly homonymous hemianopia, are frequent, and limb sensory impairments, exteroceptive or proprioceptive, affect about half of stroke survivals [4]. Successful movement also relies on efficient sensorimotor integration, so its dysfunction may also lead to alteration of posture, balance, and gait, influence the functional outcome of patients, and affect the efficacy of neurorehabilitation [5,6,7,8]. Gait patterns have been extensively analyzed and quantified in post-stroke subjects using three-dimensional Gait Analysis (3D-GA), revealing slower and asymmetric gait patterns [9], abnormal kinematics, and reduced ankle power during terminal stance [10].

The 3D-GA is widely used to provide comprehensive data on normal and pathological gait in dedicated laboratories to obtain a fine motor assessment during rehabilitation protocols or for scientific purposes. This is an important method for obtaining crucial information to determine the severity of functional limitation, to follow up the evolution over time, and to establish proper rehabilitation treatments able to reduce the impairment effects and favor the gait recovery [11]. This information is obtained by measuring the kinematics and kinetics of major body segments and joints using optoelectronic systems and force platforms in a well-instrumented laboratory and with trained and experienced personnel. Among the many relevant measures (i.e., spatiotemporal parameters, kinematics, and kinetics), spatiotemporal parameters are mainly considered in a clinical setting. These quantitatively describe the main events of gait, and thus reflect the patient’s ability to meet the general gait requirements, such as weight acceptance, single limb support, and swing limb advancement [12]. Several methods are available for measuring spatiotemporal parameters, including optoelectronic systems, which are considered the gold standard in clinical practice [13] due to their measurement accuracy.

According to the concept of ecological validity [14], alternative methods and technologies have also recently been proposed for the evaluation of spatiotemporal parameters, overcoming the typical limitations of measurements in indoor laboratory environments, such as high costs, dependency on trained personnel, and the need to wear few clothes [15,16,17]. Among these approaches, low-cost optical body tracking sensors (i.e., RGB-D cameras) have found widespread use for non-invasive analysis of human body movement in medical research, including new approaches for limb movement detection and tracking, assessment of motor capacity, action recognition, and gesture and posture classification [18,19,20,21,22]. Furthermore, several applications for rehabilitation and mobility recovery have been designed by exploiting the innovative human–computer interaction based on body movement captured by this kind of sensor, even in virtual environments [23,24,25].

Regarding gait analysis, several studies have adopted a multi-camera approach to cover a longer corridor length (up to 10 m) as in traditional 3D-GA [26,27]. This method is commonly used to overcome the maximum distance of the body tracking algorithm (which normally covers up to 4.5 m) but increases the complexity of management and calibration. Alternatively, RGB-D cameras are frequently paired with treadmills [28,29] to increase the quantity of steps available for analysis. Due to their cost and size, the implemented solutions are only feasible in motion analysis or research laboratories. However, they are not suitable for smaller domestic and unsupervised environments, where a single-camera solution on a shorter walking path would be preferable.

Some researchers have proposed a single RGB-D-camera approach in Parkinson’s disease patients to assess upper limb tasks [30], posture [31,32,33], lower limb movements [33], and Tinetti scale [34]. Single-camera approaches have been also used to evaluate gait patterns in children with cerebral palsy [35], subjects with ataxia [36], Parkinson’s disease [37], and polyneuropathy [38], or simply to analyze gait patterns in young and old people [39]. Regarding post-stroke patients, RGB-D sensors have been used to predict the risk of falls [40], to evaluate the motor function of upper limbs [41], to analyze balance recovery [42], for the rehabilitation of the upper limbs [43], and for gait analysis [44].

A validation procedure is required to evaluate the accuracy of such non-invasive devices in acquiring gait results. Studies on healthy people [39] and subjects with Parkinson’s disease [45], hemiparesis [44,46,47,48], diabetic sensorimotor polyneuropathy [49], and other neurological disorders [50,51,52,53] revealed that most of the spatiotemporal parameters of gait can be assessed appropriately. Concerning post-stroke applications, in [44] the authors compared the reliability of different gait parameter estimation methodologies to distinguish healthy and post-stroke subjects. In [46], the authors considered stride length as an index of disease severity correlated with standard clinical scales. In [48], the study aimed to investigate the validity of gait parameters versus standardized clinical tests and their ability to assess the risk of falls in people after stroke. However, few validation studies [54,55] have been conducted on post-stroke subjects, comparing the parameters obtained from a single-camera solution and a gold standard (i.e., an optoelectronic system), especially regarding gait [47]. In our approach for reliable monitoring over time, we included this comparison to assess even subtle differences in the gait of post-stroke subjects. Other validation studies on gait that involved RGB-D sensors are available on healthy subjects, but use a multi-camera approach [26] or a different gold standard system (GAITRite, CIR system Inc., Franklin, NJ, USA, Sparta, N) [56], and on children with cerebral palsy [35].

In this context, the present study proposes a vision-based system built around a single RGB-D camera to analyze gait patterns and spatiotemporal parameters over a short walkway. It is not intended to provide precise clinical diagnoses or to become a substitute for 3D-GA. Its aim is to estimate gait parameters using simple and non-invasive technologies that can be a straightforward method to monitor the alteration or improvement of gait in post-stroke subjects, for example, as a consequence of rehabilitation treatments. It is particularly notable from the clinical perspective that our system would also make it possible to provide remote monitoring and rehabilitation solutions for gait that are suitable for home settings and user-friendly, thus overcoming the main constraints of gait analysis in the laboratory and allowing assessments in the real contexts of life, where 3D-GA is impractical. For this purpose, however, it is necessary to establish the level of accuracy, limitations, and feasibility of a single RGB-D camera approach. This study aims at comparing a subset of spatiotemporal parameters with a gold standard system on a shorter walking path that is appropriate to the spaces available in home environments. This is the preliminary step to provide an accurate, contactless, and easy-to-use methodology for analyzing gait at home. Finally, considering the perspective of employing the proposed system for monitoring and rehabilitation in a home environment, the Timed Up and Go (TUG) test was chosen and included in the experimental protocol as a first step to analyze the correlation between the estimated gait parameters and a functional reference test in the clinical evaluation of post-stroke individuals.

## 2. Materials and Methods

### 2.1. The RGB-D System: Hardware and Software

**Figure 1:**
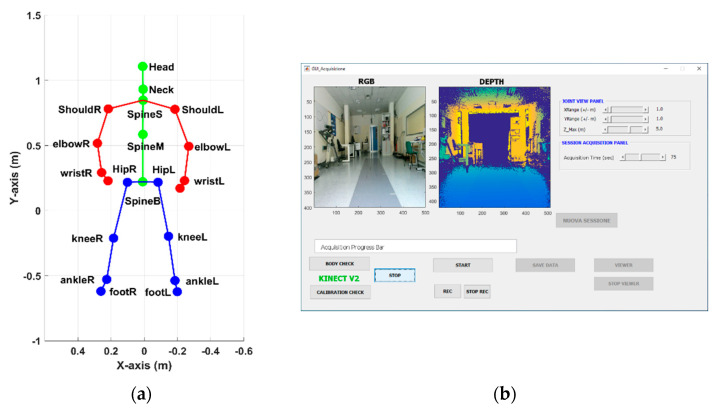
(**a**) Skeletal model and joint positions during the preliminary standing phase; (**b**) main GUI of the acquisition software to control the RGB-D system.

The RGB-D optical sensor used in this study, specifically the Microsoft Kinect© v2 camera (Microsoft Corporation, Redmond, WA, USA), is the core of a vision-based system suitable for acquiring and analyzing gait patterns and other motor activities [33]. It connects to a Windows 10 laptop or mini-pc through a dedicated USB port. A monitor or a TV screen, which displays the user interface and provides visual feedback of the body movements, completes the system. The RGB-D sensor provides synchronized color, infrared, and depth streams at a maximum frame rate of 30 frames/second, with a maximum resolution of 1920 × 1080 pixels for color, and 512 × 424 pixels for depth and infrared streams. The frame rate allows the human body motion to be captured in real-time up to a distance of 4.5 m. The availability of several streams enables the development of ad hoc tracking algorithms based on computer vision techniques [57]. The body tracking algorithm captures movements in real time by detecting body patterns in depth maps estimated using time-of-flight technology and mapping specific body regions on a skeletal model consisting of 25 joints [58]. Each joint refers to an anatomical body point, and a three-dimensional position represents it according to the RGB-D sensor reference system. An example of the skeletal model during gait is shown in Figure 1a.

The vision-based system is equipped with custom-written software that consists of ad hoc MATLAB^®^2018 scripts (Mathworks Inc., Natick, MA, USA). Some scripts interface with the RGB-D sensor to retrieve and save all the raw data available every 30 msec, including images, skeletal model, and joint information. In particular, skeleton and joint data are used for the analysis procedure. Other scripts implement the graphical user interface (GUI) that helps the operator (e.g., clinician, therapist, or technician) to easily perform data acquisition. The main GUI (Figure 1b) consists of graphical objects, buttons, and panels, to configure the motion capture system according to clinical needs (e.g., set the maximum duration of gait). In addition, the same GUI activates the proper sequence of operations to correctly acquire the patient’s gait: first, to check the proper functioning and positioning of the sensor (through color and depth streams); then, to start and stop the data recording; and, finally, to display the data recorded, specifically the skeletal model, to qualitatively verify the correctness of the acquisition. Once the gait acquisition is complete, the collected information is saved “per frame” as MATLAB files in the session folder created automatically during the initialization phase. MATLAB files are used by the data analysis procedure to estimate spatiotemporal parameters.

### 2.2. The 3D-Gait Analysis

Participants were involved in standardized gait analysis trials (3D-GA). They were recorded at a sampling frequency of 50 Hz by means of an optoelectronic motion capture system consisting of six cameras (VICON, Oxford Metrics Ltd., Oxford, UK) and two force platforms (Kistler, Winterthur, Switzerland) while walking barefooted along a 10 m walkway at a self-selected comfortable speed.

Prior to the experimental tests, the following anthropometric data were collected: height, body mass, anterior superior iliac spine distance, pelvis thickness, knee and ankle width, and leg length. Then, spherical retro-reflective passive markers were placed on the individuals’ skin at specific landmarks according to the protocol proposed by Davis [59]. Markers were placed over C7 and sacrum, and bilaterally on the anterior iliac spines, greater trochanter, femoral epicondyle, femoral wand, tibial head, tibial wand, lateral malleolus, lateral aspect of the foot at the second metatarsal head, and the heel. Before performing the gait test, a standing trial was also recorded for each participant to evaluate the standing posture. They were instructed to maintain an upright standing position for 5 s, with open eyes, focusing on a 6 cm black circle positioned at the individual’s horizontal line of sight at a distance of 1.5 m. Subjects placed their arms at their sides and their feet were in a comfortable position. The optoelectronic system was calibrated before starting each acquisition session to guarantee the proper tracking accuracy for the calibrated working volume and the correct estimation of 3D coordinates of passive markers.

Because this study focused only on the comparison of spatiotemporal parameters, other quantities estimated during 3D-GA (in particular, angles, moments, and powers) were not considered for data analysis, although these data were available.

### 2.3. Setup

An experimental setup was defined to allow a fair comparison between the two sets of spatiotemporal parameters estimated by the systems. The optical sensor is fixed on a tripod, approximately 1m above and perpendicular to the ground, and is positioned at the end of the walking path. The tripod ensures the device stability and allows an easy correction of the angular orientation along the three reference axes. This setup allows for gait capture from the front, i.e., while the subjects are walking towards the RGB-D sensor. The frontal view ensures the maximum accuracy in depth estimation and, consequently, in body tracking. The RGB-D sensor shows greater accuracy along the central vision cone, whereas the vertical and lateral scattering of light pulses reduces the accuracy in the corresponding areas [60]. In addition, a loss of body tracking accuracy has been highlighted in other configurations, for example, in rear [61] or lateral [27] views, or in different viewpoint directions [60].

**Figure 2:**
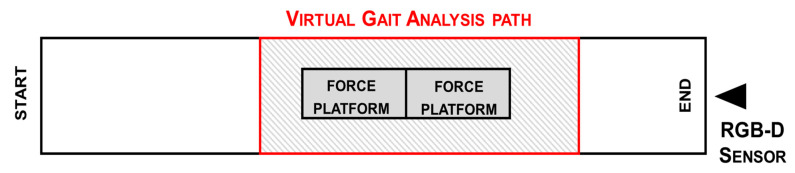
Scheme of the experimental setup: position of RGB-D sensor and gait analysis path (red) inside the walkway to ensure total body motion capture and analysis.

The body tracking is automatically activated approximately 4.5 m from the optical sensor when a subject enters the camera’s field of view. Thus, we defined a virtual gait analysis path (VGAP) across the mid-zone of the 10 m walkway and around the force platform, to ensure full body motion capture during gait by the RGB-D system and, simultaneously, by the 3D-GA. The start of the VGAP is fixed at about 4.2 m from the optical sensor, and the end is at a distance of about 1.5 m. In this area, the depth accuracy is between 2 and 4 mm at the end of the working volume, i.e., beyond 4 m from the sensor [60]. The accuracy and robustness of the body tracking were previously verified using a similar configuration [33], in which the comparison with the gold standard system showed a high correlation with the 3D trajectories from which the linear and angular parameters are estimated. The VGAP is about 2.7 m long. Although the walking zone is relatively limited with respect to the overall walkway, it has been shown that it is sufficient to detect at least one full stride per leg based on the severity of gait impairment [62]. However, the shorter gait path is more suitable for home environments, which was the goal of this study. In addition, setting the starting point outside the VGAP allows subjects to enter the analysis area at their normal pace. Figure 2 shows the position of the VGAP with respect to the walkway and the 3D-GA system.

### 2.4. Acquisition Protocol

The acquisition protocol consists of some preliminary procedures before starting data collection. First, passive markers are applied to the subject’s skin according to anthropometric measures. Then, the subject has to complete practice gait trials along the walkway, to verify the visibility of all the markers by the 3D-GA system and the subject’s familiarization with the experimental procedure.

After preliminary trials, the subject was asked to maintain an upright position for 5 s at the opposite end of the walkway with respect to the RGB-D sensor. Then, the subject was asked to walk, at a normal pace, straight and forward until reaching the RGB-D sensor. Thus, the subject entered and exited the VGAP at the best walking ability, consistent with the disability.

As soon as the subject enters the sensor’s field of view, the body tracking algorithm starts generating the skeletal model and estimating the 3D position of the 25 joints. At the same time, the RGB-D system starts recording all information related to the skeletal model, color, and depth frames, which are saved for any offline clinical supervision. When the subject leaves the sensor’s field of view, body tracking and data recording stops automatically. Data acquisition by the two systems starts simultaneously, in order to compare gait parameters.

The acquisition protocol includes five trials for each subject to ensure reproducibility of the results and to select the best data for the subsequent analysis procedure. Between each trial, the acquisition protocol establishes a short pause to allow subjects to recover from fatigue.

### 2.5. Participants

A cohort of 11 post-ischemic patients was recruited for the study (side: 4 with left and 7 with right hemiparesis; gender: 3 females and 8 males; age: 53.3 ± 13.9 years; time since first major stroke event: 52.32 ± 18.48 months). According to the classification of Bamford [63], all patients showed the features of partial anterior circulation infarcts (PACI), with only partial impairment of the anterior cerebral circulation. Nine patients presented sensory deficits, whereas dysphasia or visuospatial disorders were present in four and five patients, respectively. The participants were selected at the Division of Neurology and Neurorehabilitation, San Giuseppe Hospital, Istituto Auxologico Italiano, Piancavallo (Verbania), Italy. Recruitment criteria included: independent walking for 10 m without aids or the assistance of another person; and ability to understand the instructions for performing the gait analysis test. The exclusion criteria were cognitive impairment with Mini-Mental State Examination (MMSE) <26; and history of other neurological or musculoskeletal disorders unrelated to stroke.

Prior to gait analysis, patients were examined using the Timed Up and Go (TUG) test. The TUG test is a performance-based measure of functional mobility that was initially developed to identify mobility and balance impairments in older adults, and requires motions of balance, sit to stand, walking, and turning. It measures the time to stand up from a chair, walk three meters, turn around, return to the chair, and sit down again [64,65]. The TUG test was included in the experimental protocol because it is a simple and validated tool for clinical evaluation of gait function, and, from the rehabilitation perspective, it is considered a sensitive and specific measure to evaluate risk of falls among older adults. In this study, TUG results were correlated with the estimated parameters obtained with our system, in order to provide additional information to clinicians using a familiar reference.

The selection criteria were established also considering the potential end-users of the system proposed in this study. For this reason, we enrolled only hemiparetic subjects previously evaluated and selected for standard rehabilitation in hospital settings and who could benefit from continuing prolonged neurorehabilitation at home.

The study was approved by the local ethics committee in accordance with the 1964 Helsinki declaration and its latest amendments. All participants were volunteers and gave written informed consent before being enrolled for the study.

### 2.6. Data Processing

The data analysis for the 3D-GA acquisition of 3D trajectories of passive markers relies on detecting the first heel impact event for each foot on the force platform. Previous and subsequent similar events were identified based on the kinematic attitude of the first event detected. Two or three complete gait cycles were considered, depending on the participant’s motor disability, overlaying the VGAP defined for the analysis of RGB-D data. The data analysis procedure for 3D-GA was performed using the software analysis available with the optoelectronic system.

By comparison, the data analysis for the RGB-D system is based on the 3D trajectories of the joints, and, for this purpose, dedicated software was developed using MATLAB. The analysis procedure involves a preprocessing phase. The 3D trajectories are resampled using the MATLAB cubic interpolation functions to remove the slight fluctuations in the device’s frame rate (around 30 FPS on average) and to align the time baseline of the two systems. Then, a Butterworth low-pass filter (third order, 10 Hz cut-off) is applied to the resampled data to reduce noise without excessive smoothing of trajectories, although other filtering methods can be considered [66]. Then, an ad hoc algorithm performs the step segmentation phase, in which the ankle joints were used to estimate steps and gait cycles. Due to the high instability and low tracking accuracy of foot joints, ankle joints were preferred [67].

**Figure 3:**
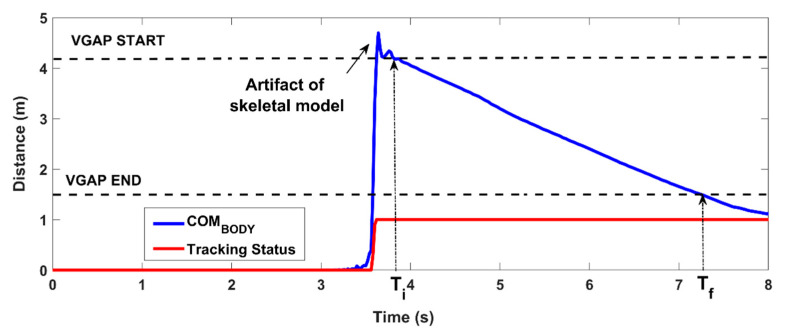
Estimation of the time window of analysis: the COM_BODY_ is used to determine when the subject enters the VGAP (T_i_) and when the subject leaves the VGAP (T_f_). The analysis time window is between T_i_ and T_f_.

The body center of mass (COM_BODY_) is calculated using the skeletal model as described in [33], and its 3D distance (COM_D_) from the RGB-D sensor is used to establish the time window for the analysis. The initial time (T_i_) is the time in which the body enters the VGAP (COM_D_ < 4.2 m), and the final time (T_f_) is the time in which the body leaves the VGAP (COM_D_ < 1.5 m). There is a slight delay between the activation of the body tracking (i.e., when the body enters the field of view) and the time T_i_. This allows any initial artifacts of the skeletal model to be excluded that may interfere with the automated analysis, as shown in Figure 3.

The 3D trajectories of the left (ANK_L_) and the right (ANK_R_) ankles are used by the step segmentation algorithm to estimate gait parameters for each leg within the time window established for the analysis.

**Figure 4:**
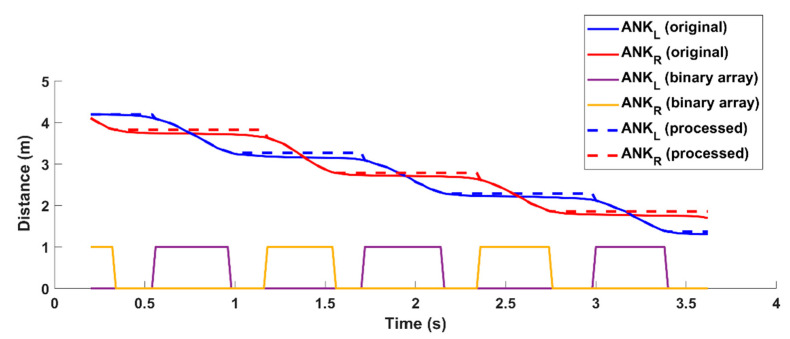
Estimation of binary arrays from the trajectories of ankle joints within the time window used for the analysis. Solid red and blue lines refer to the original trajectories of ankles; dotted red and blue lines refer to the processed trajectories with constant Z component during the stationary condition. The graph also shows the solid yellow and purple square lines corresponding to the two binary arrays to highlight the “stationary” (0 values) and the “in movement” (1 values) periods.

The algorithm works on the ankle depth values (z-axis) to perform binary thresholding of the z-component to detect each step. The threshold is set to 2 cm; this value was determined through preliminary tests on healthy volunteers by simulating different gait patterns. When the z-component is almost constant (i.e., the difference between two consecutive z-values is less than the predefined threshold), the algorithm considers this condition corresponds to the ankle’s “stationary” period. Conversely, when the z-component shows significant variations (i.e., the difference between two consecutive z-values is greater than the predefined threshold), the algorithm associates this condition with the ankle’s “in movement” period. In practice, the algorithm generates a binary array per leg; the value 0 refers to the “stationary” condition and the value 1 refers to the “in moving” condition. To reduce the slight fluctuations in depth, the z-value of the ankle is forced to the initial value and held constant throughout the “stationary” period. Figure 4 shows the processing of the z-components of the ankles and the generation of the binary arrays.

**Figure 5:**
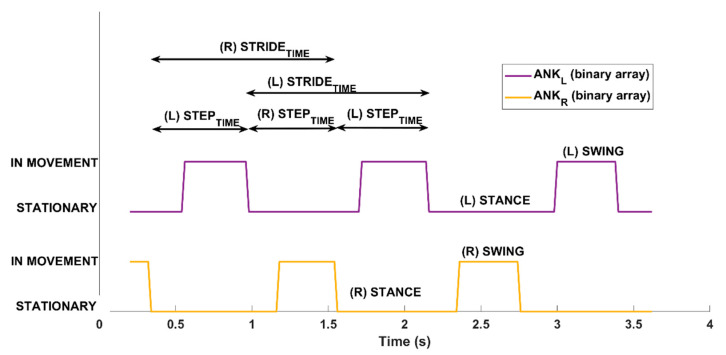
Step segmentation algorithm: Automatized analysis from the binary arrays within the time window used for the analysis to estimate gait parameters for left and right legs. The “stationary” and “in movement” periods for the two legs are displayed. In addition, the figure shows some of the steps for left (L) and right (R) legs from which both spatial and temporal information are evaluated.

The two binary arrays are then used to extract meaningful information on gait patterns corresponding to traditional spatiotemporal parameters of gait analysis [39,56,68]. Figure 5 shows the result estimated by the analysis procedure from the binary arrays, i.e., the sequence of the left and right steps, and some temporal values.

### 2.7. Estimation of Gait Parameters

From the data collected by the RGB-D and the 3D-GA systems, a subset of the traditional spatiotemporal parameters and the 3D estimated trajectory of the body center of mass (COM_BODY_) were computed and analyzed. Traditional gait analysis produces many parameters, but, in general, only a limited number are considered in clinical practice. The current study aimed to focus on a minimum set of spatiotemporal parameters which, however, provide immediate evidence of gait anomalies [69].

An interesting aspect related to gait impairments in post-stroke subjects is the oscillations of the body center of mass during gait [70,71]. It is probable that lateral oscillations of the body are observed in the case of greater difficulty in walking to correctly counterbalance the weight of the body.

To analyze this feature, the 3D-GA system estimated the trajectory of COM_BODY_ starting from the 3D trajectories of the passive markers [72]. Then, the peak-to-peak displacements, or “excursions” or “sway”, of COM_BODY_ along the medio-lateral (ML) and vertical (V) directions of the gait were computed. Alternatively, the RGB-D system considered the hip midpoint trajectory (COM_HIP_) for the analysis, estimated from the HIP_L_ and HIP_R_ joints of the skeletal model. In this case, the peak-to-peak displacements, or “excursions”, of COM_HIP_ along the medio-lateral (ML) and vertical (V) directions of the gait were computed and then compared with the corresponding parameters estimated from COM_BODY_.

The basic events detected by the two systems for the extraction of the gait parameters were not exactly the same due to the different 3D positions of markers and joints, and different processing algorithms. Table 1 presents the estimated parameters, their meaning, and the estimation rules for RGB-D and 3D-GA systems.

All the spatiotemporal parameters were calculated for each trial as the average values of all detected events, with the exception of the cadence, which was computed considering the total number of steps within the VGAP. The parameters relating to the hemiparetic and the non-hemiparetic leg were estimated separately. The parameters related to center of mass (COM) were computed considering the maximum excursions of body within the VGAP.

### 2.8. Statistical Analysis

Three consistent trials of the five trials performed were selected and analyzed for each participant to estimate spatiotemporal and COM parameters. The selected trials were devoid of artifacts, hesitations, and errors in entering or exiting the VGAP, carried out in a straight line while walking, and performed at the best walking ability, as reported by the patients.

First, the Kolmogorov–Smirnov test [73] was used to verify the possible normal distribution of the estimated parameters. Because the normality hypothesis was not satisfied, we considered the median interval and the corresponding quartile for all the parameters. Then, the Wilcoxon test [74] was used to verify the statistical difference between the parameters estimated by the two systems. In this study, the absence of a statistical difference between the parameters estimated by the two systems indicated their congruency and compatibility.

To evaluate the level of agreement between the 3D-GA and RGB-D systems, Spearman’s correlation coefficient [74], intra-class correlation (ICC), and Bland–Altman plots [75] were considered.

To address the non-normal distribution of the parameters, we used Spearman’s nonparametric rank correlation. Spearman’s correlation is commonly used to assess the strength of the monotonic relationship between two variables. Spearman’s correlation coefficients range between −1 and 1. A value equal to 1 denotes an excellent positive correlation between two measures; a value equal to −1 denotes an excellent negative correlation between them; a value near zero indicates a negligible correlation (positive or negative). For in-between values, the conventional approach considers values greater than 0.7 as indicators of strong correlation, values between 0.4 and 0.7 as indicators of good correlation, and values between 0.1 and 0.4 as indicators of weak correlation [76]. The initial hypothesis was to detect a high correlation between the parameters estimated by the two systems, and in the same direction (positive correlation). In addition, we also used Spearman’s correlation to evaluate the correlation of the estimated parameters with respect to TUG. Because higher execution times depend on significant walking impairments, parameters estimated by the gait analysis with 3D-GA and RGB-D systems were expected to show a positive correlation with the TUG test.

To evaluate absolute agreement, we used ICC [77]. ICC is commonly used to assess the consistency and reproducibility of objective measurements of the same quantity made by different observers or systems. ICC has wide application in medical research, for example, as a reliability index of experimental methods [78]. ICC is a positive value that varies between 0 and 1. A value less than 0.5 indicates a poor reliability or consistency of the measures; a value between 0.5 and 0.75 indicates a moderate reliability or consistency; a value between 0.75 and 0.9 indicates a good reliability or consistency; a value above 0.9 denotes an excellent data reliability or consistency [79]. In this case, ICC was used to check the correlation between the parameters of the 3D-GA and RGB-D systems. The initial hypothesis is a high correlation, that is, absolute agreement, between the two classes (i.e., systems). Bland–Altman plots are commonly used to assess the agreement between two or more instrumental measurement methods [77] using a graphical representation of the differences. Thus, we included this test in our statistical analysis. This test was used to check whether the RGB-D system underestimated or overestimated parameters compared to the gold standard 3D-GA. The initial hypothesis was that the estimated parameters were within the Limits of Agreement (LoA). The LoA interval is established as the “mean difference ± 1.96 * standard deviation”, according to the pre-established confidence interval (95%). For all the statistical tests, probabilities below 0.05 (*p* < 0.05) indicated rejection of the null hypothesis.

Finally, the accuracy of the measurement (or percentage measurement error) was calculated for each parameter according to the formula ACC_%_ = |Value_REF_—Value_CUR_|/|Value_REF_|, where Value_REF_ is the reference value measured by the 3D-GA system and Value_CUR_ is the current value measured by the RGB-D system.

## 3. Results

### 3.1. Statistical Analysis and Correlation Results

The statistical analysis relies on three selected consistent trials (among the five performed) of each participant (eleven post-stroke individuals) captured simultaneously by the two systems. For the estimation of the spatiotemporal parameters, the hemiplegic and non-hemiplegic sides were analyzed separately. In contrast, the COM parameters were estimated as a single value for each trial.

The statistical analysis using the Wilcoxon test revealed a significant difference between the two systems only for step width (*p* < 0.05), indicating disagreement between the two systems for this parameter. In contrast, all other parameters did not show statistical differences between the two groups (*p* ≥ 0.05), indicating agreement between the two systems. Table 2 shows the median and the first quartile values for each parameter: Only step width was statistically different between the two systems, as reported in the column with the *p*-values and the effect sizes. The effect size is small or negligible for all the parameters that do not show a statistical difference.

For the analysis of the agreement between the two systems, the Spearman’s correlation coefficient and intra-class correlation (ICC) were considered, applying a significance level of 95% (i.e., *p* < 0.05) for both; Table 3 shows the results of the statistical analysis.

It is important to remark that the correlation analysis provides the same indication about the estimated parameters.

**Figure 6:**
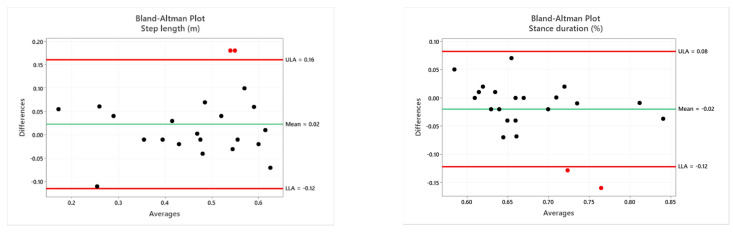

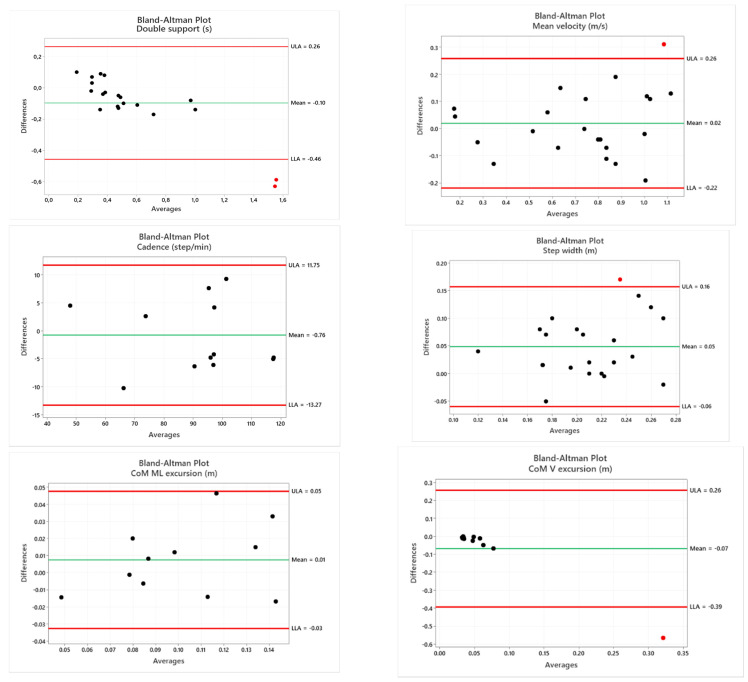
Bland–Altman plots of the mean of instrumented 3D-GA and the RGB-D systems plotted against the difference between the two methods. For all spatiotemporal parameters, 22 values are reported because they are considered separately for the right and left limb, with the exception of cadence [12] and parameters related to COM.

The Bland–Altman analysis is a graphical method of evaluating the agreement between two measurement techniques and an easy means to verify where 95% of the differences fall [80]. The horizontal lines correspond to the mean difference and the LoA. The LoA interval is established as the mean difference ± 1.96 * standard deviation. The differences between two paired values are drawn as y-values. For almost all parameters, 95% of the differences fall inside the LoA. Figure 6 shows the Bland–Altman plots for all the parameters estimated. Bland–Altman plots are scatterplots of the mean between the RGB-D system and instrumented 3D-GA plotted against the difference between the two methods. The mean of the two measurements is reported as the x-value and the difference between them is reported as the y-value. Because most of the points fall within the interval given by the LoA, the Bland–Altman graphic analysis indicates the good association between the two measurement systems. The red outliers refer to the same patient’s session where only the first three trials were correctly performed. The others were discarded because they were not compliant with the experimental protocol (the patient could not continue due to excessive fatigue). The patient’s test session was also characterized by slight instability in the motion tracking, probably caused by a partial interference of the ambient light conditions compared to the trials performed by the other participants. In this session, it was necessary to change the environmental light to ensure better visibility for the patient. This condition probably affected the tracking of the joints/markers in some walkway areas, causing a more significant difference between the two systems and in the estimated spatiotemporal and COM parameters, particularly the vertical sway, which shows greater disagreement than the other trials.

### 3.2. Gait Patterns

**Figure 7:**
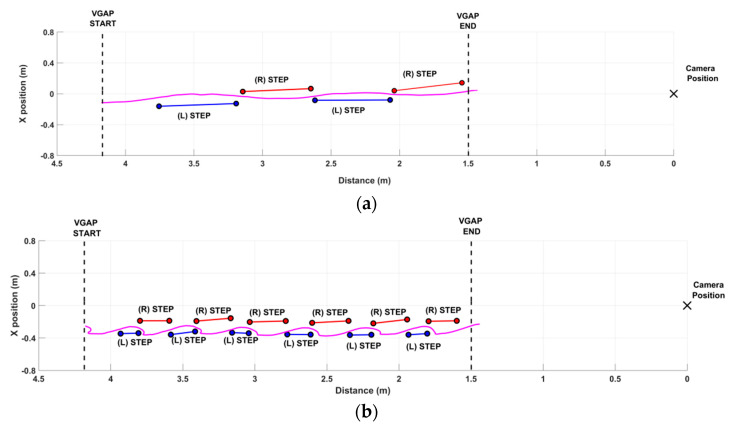
Examples of gait analysis with the step segmentation algorithm. (**a**) Example of a good performance: two steps for both the legs within the VGAP. The COM_HIP_ trajectory (solid magenta line) shows small excursions, denoting relatively good body control during walking. (**b**) Example of a significantly impaired performance, characterized by shorter steps and higher body sway, indicating the attempt to compensate for impairment during walking.

Figure 7 shows the performance of two participants as a result of the step segmentation algorithm. The dotted black lines delimit the VGAP zone. The black cross indicates the position of the RGB-D sensor at the end of the walkway. The x values of the graph correspond to the 3D distance from the sensor over time (the x-axis of the graph corresponds to the z-axis of the RGB-D sensor, and the y-axis to the x-axis of the sensor).

Figure 7a shows an example of a good gait pattern. The VGAP is walked quickly and with a small number of steps, indicating a good pace. This subject showed no particular impairments during walking. This behavior is also confirmed by the low oscillations of the center of mass, as visible from the trajectory of the COM_HIP_ (solid magenta line). On the contrary, Figure 7b shows an example of an impaired gait pattern. The VGAP is walked slowly and with many short steps, denoting considerable difficulty in walking. This subject usually walked supported by a stick that was not used. During the trial, the therapist walked sideways to promptly intervene in case of excessive difficulty and fatigue. Many shorter steps characterized the performance, as also detected by the step segmentation algorithm. The anomalies in gait pattern were also confirmed by the analysis of the COM_HIP_ trajectory (solid magenta line), showing higher lateral sway to achieve better dynamic balance as a compensatory strategy for gait disorders.

In addition, Table 4 shows the values of spatiotemporal parameters estimated for the paretic and non-paretic limbs by the RGB-D system for the first (#1) and the second (#2) gait patterns graphically represented in Figure 7a and Figure 7a,b, respectively. Cadence and COM parameters refer to the general gait pattern and are not associated with a specific side of the body; thus, we omit them from Table 4. In both cases, the system detects a difference between the paretic and the non-paretic sides. As expected, the paretic side is characterized by lower step length and velocity than the non-paretic side, whereas the stance phase and double support duration show no difference between the two sides. In addition, spatiotemporal parameters significantly degrade with increasing gait deficit (#2), with the except of step width, which is not affected. This quantitative result confirms the qualitative evidence from Figure 7a,b. This also occurred for the cadence, which is lower when gait impairment increases (estimated cadence is 99.17 steps/min for #1 and 45.77 steps/min for #2). Regarding the COM parameters, the ML sway highlights the gait deficit severity (estimated ML sway is 0.088 m for #1 and 0.126 m for #2), whereas the V sway appears not to be affected (estimated V sway is 0.038 m for #1 and 0.042 m for #2).

Finally, the estimated spatiotemporal and COM parameters were correlated to the TUG test performed by each participant before starting the gait analysis trials. Table 5 shows the correlation coefficients for the two systems. Results indicate that all spatiotemporal parameters, with the exception of step width, strongly correlate with TUG times, as initially expected. In addition, the sign of each coefficient indicates the type of relationship with the TUG test. In particular, step length, mean velocity, and cadence exhibit an inverse relationship with the TUG test; the TUG time increases in the case of shorter steps or lower speeds. On the contrary, stance duration and double support duration show a direct relationship with the TUG test; the TUG time increases in the case of longer stance or double support phases. Step width probably does not affect the total duration of the TUG test, so the estimated correlation is negligible. The COM parameters show poor correlation with the TUG test. The ML and V excursions characterize the gait patterns, but probably do not have the same relevance as the spatiotemporal parameters in the TUG test.

**Figure 8:**
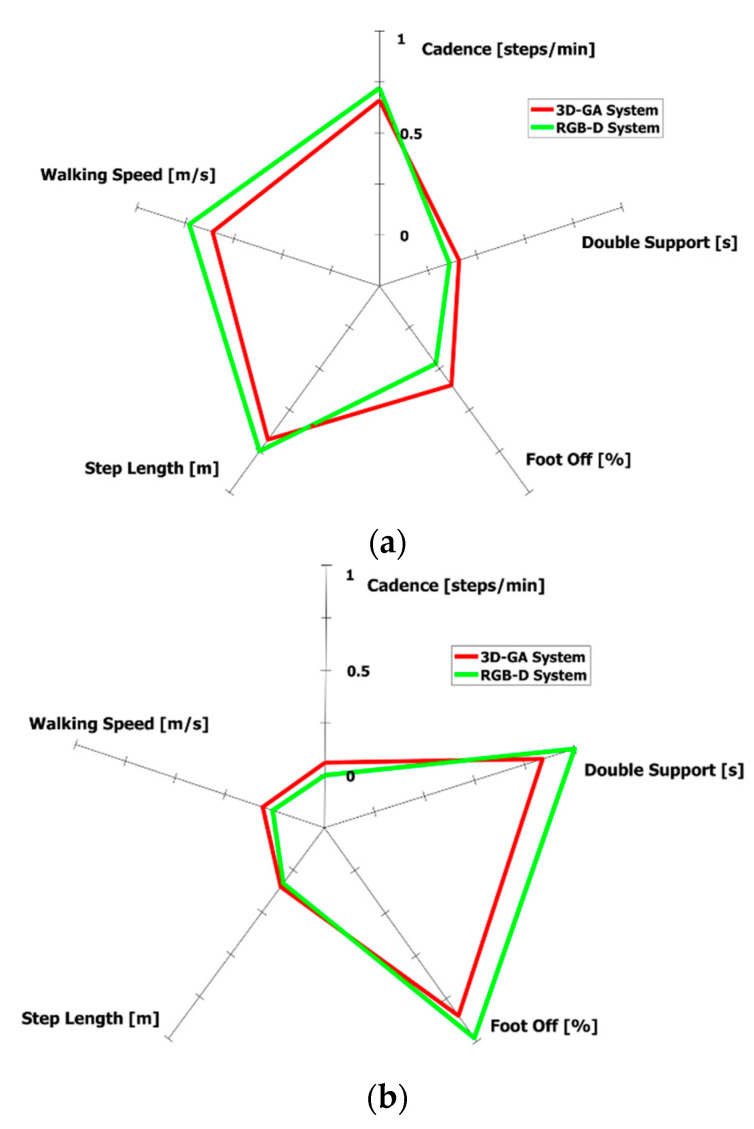
Example of radar charts on the estimated spatiotemporal parameters. (**a**) Radar chart related to good gait performance (Figure 7a). (**b**) Radar chart related to impaired performance (Figure 7b).

A graphical representation, in the form of a radar chart, is specifically generated by our system and is intended to provide an immediate, effective, and easy-to-compare visual indication of the gait performance. Only the most correlated spatiotemporal parameters were considered, according to Table 3. To avoid the different scaling of parameters, we applied the min-max normalization by considering the maximum and minimum values of each parameter in all the participants’ trials. In this manner, each parameter value was able to be scaled in the [0–1] range to generate a clear radar chart. Figure 8 shows the radar charts related to Figure 7a,b.

## 4. Discussion

This study aimed to verify whether an RGB-D sensor could estimate spatiotemporal parameters, typical of the standard gait analysis, during level walking on a short path performed by post-stroke subjects. For this purpose, the walking abilities of a small cohort of subjects with unilateral hemiparesis were analyzed simultaneously using a single-camera approach and an instrumented 3D-GA. First, we defined an experimental setup and a virtual gait analysis path (VGAP) to analyze gait patterns. The body tracking algorithm provided by the RGB-D sensor (specifically, a Microsoft Kinect v2) allows the subject’s gait inside the VGAP to be captured via a skeletal model consisting of 25 joints. It is noteworthy that the position of the RGB-D sensor and the length of the VGAP are both compatible with the limited spaces in domestic scenarios. A step segmentation algorithm was then implemented to analyze the 3D trajectories of joints, specifically, ankle joints, to identify each left and right step during gait. A subset of the most relevant spatiotemporal parameters of standard gait analysis was estimated from this data. In addition, this study also included the analysis of the medio-lateral and vertical sways of the body center of mass (COM) during the gait, as associated with an attempt to compensate for any gait imbalance when walking. A small group of post-stroke subjects was recruited to preliminarily verify the investigation hypothesis and the reliability of the experimental system and its setting.

Preliminary results indicate no statistical differences between the spatiotemporal and COM parameters estimated by the two systems. Moreover, relevant correlation values were found for all estimated parameters that move in the same direction, as indicated by the direct relationship (positive correlation coefficients). The only exception was the step width, for which the close position of the feet could underestimate the distance on the x-axis of the RGB-D sensor (associated with the width of the walkway), thus producing a lower correlation. According to the common interpretation of correlation values (as specified in Section 2.8), all of the parameters showed strong ICC correlation and good reliability (or consistency) between the two systems, with the exception of step width, which showed a weak correlation and poor reliability, as reported by the first two columns of Table 3. In addition, all the Spearman’s correlation coefficients were positive, denoting that measurements were in the same direction (positive relationship) and predicted the same behavior. Regarding ACC_%_, the highest value was related to the step width. This value, combined with the low level of correlation, denotes a discrepancy between the measurements of the two systems. The ACC% values were also relatively high for the double support duration and V sway, but showed a strong and good correlation, respectively. For double support duration, this trend may be due to the different methods of event detection from which the parameter was estimated; for V sway, it may depend on the different estimation of COM. The same behavior, although less relevant, was found for the step length and cadence parameters.

Overall, the analysis of the data collected using the RGB-D system confirmed that it estimates the same parameters measured by 3D-GA system, as defined in Table 1. This is the main finding of our study. Nevertheless, some measurements may differ slightly due to the different positions of the reference points for the two systems (3D markers and joints of the skeletal model) or, more probably, to the different algorithms that evaluate gait parameters. Consequently, this may impact the agreement analysis between the two systems. In addition, this makes it difficult to compare results directly with other studies. Another difficulty could derive from using different correlation methods (for example, ICC, Spearman’s correlation, Pearson’s correlation), as found in several studies in the literature. For example, [47,56] reported a slightly higher agreement than our results for mean velocity and step length, but used Pearson’s correlation, whereas [26] indicated a slightly higher agreement for cadence and mean velocity. Step width shows the lowest correlation in [26], as in our findings, but this appears to be in contrast with other validation studies [29,62]. Conversely, correlation and ICC values for step length are similar to those estimated in [81]. For stance duration, the correlation is in line with stride time and stance time in [29], as it is expressed as a percentage of stride time, whereas double support appears to be more correlated in our study. In addition, the range of spatiotemporal parameters in this study is similar to the normative gait parameters for the age-matched individuals in [48], which also show a significant correlation with traditional clinical tests, with the exception of step width. This supports our results on the correlation between the spatiotemporal parameters and the TUG test, as expected (Table 5). Regarding parameters related to the body center of mass, the preliminary analysis of trajectories along the mediolateral direction appears to confirm higher incidences of poor walking, as indicated in [71]. However, this qualitative analysis (Figure 7) needs to be more closely explored using a larger sample size of individuals.

From the clinical perspective, our results show that data obtained by the single RGB-camera system provide an adequate estimation of a subset of traditional gait parameters that is comparable with more complex optoelectronic systems that are considered the gold standard in movement analysis. The accuracy should clearly be further evaluated with larger samples and broader levels of gait impairments. However, it is important to note that the system we propose is not intended as a substitute for the gold standard 3D-GA, but to be used as an intermediate tool for gait analysis in those environments where the gold standard is not applicable (i.e., in home settings).

Our system is not intended for specific diagnostic purposes, but may provide accurate information relating to the gait patterns, integrating clinical evaluation, in different rehabilitation contexts. It may permit the initial preliminary assessment of gait parameters in controlled environments (e.g., laboratory settings). The progress can then be remotely monitored at home in practical life contexts or in other unsupervised settings, while performing rehabilitation protocols. This approach is not possible using 3D-GA. However, in contrast, our system would enable this approach to be extended to a wide number of patients. It is also important to note that our system could provide a finer evaluation of motor patterns compared to clinical judgments, thereby detecting changes not yet recognized by clinicians or scales. These aspects are important to promptly modulate and personalize the motor tasks during rehabilitation sessions at home, reducing the need of hospital evaluations.

Rehabilitation settings are, in general, less demanding regarding accuracy requirements, especially in in-home environments. However, the possibility of detecting changes in gait patterns with an affordable, automated, and user-friendly technological solution is a priority, and the system we propose is adequate for this aim. Similarly, errors in assessing step width may be an issue for a finer analysis and diagnostic purposes, but cannot be decisive in this context. Monitoring gait parameters using a simple solution may be helpful in unsupervised settings, thus allowing evaluation to take place outside of the laboratory and overcoming the limitations of gait analysis in laboratory settings. Regarding the 3D-GA system, the proposed solution has the advantage of acquiring data without undressing patients for marker placement, which often represents a limit, especially from a psychological perspective. In addition, the experimental setting is simple, expert operators are not required, and the patient’s preparation is effortless and quick. It is important to underline that traditional 3D-GA also relies on wearing very few clothes, which causes anxiety or embarrassment to the patients, as previously demonstrated [15,17]. On the contrary, the use of contactless and non-invasive RGB-D sensors does not require marker placement or the wearing of few clothes to measure gait parameters.

Considering all these advantages, the solution described in our study may be applied to long-term neurorehabilitation programs for post-stroke subjects, including in domestic and unsupervised environments, in order to evaluate its effectiveness for gait patterns [82,83,84,85]. Clinicians may continue to use the clinical scales and tests that are familiar to them (such as the TUG test), but can also rely on an automated assessment of gait, consistent with the scales, which is able to provide significantly more information and allow detection of finer changes.

The use of outpatient and home settings may improve patient’s health and family care, and permits the reduction of National Health System (NHS) expenses, specialist visits, and repeated hospitalizations, thus providing advantages in terms of rehabilitation and economic resources. More recently, COVID pandemic restrictions have also clarified that monitoring and rehabilitating pathological subjects in home settings may become a valuable and desirable means to continue the health care of frail subjects. Consistent with this aim, the system we describe may allow the integration of gait analysis with exergames and ecological exercises to stimulate more intensive and personalized training rehabilitation activities that could improve gait strategies and reduce global impairment.

Some aspects of this study require further investigation. For example, the number of participants is limited and should increase to achieve a more robust characterization of gait patterns and parameters. The relatively small sample size limits the generalization of clinical and statistical findings. However, the preliminary results are encouraging and demonstrate the feasibility of a simple solution for gait analysis on a short walking path using a single RGB-D sensor.

Thus, further investigations may improve the validation of the solution, including the analysis of angular parameters and assessment of postural attitude during the gait. However, in subjects with hemiparesis, the gait is often characterized through few relevant spatiotemporal parameters that provide significant information about gait strategies and the quality of mobility for clinical and rehabilitation purposes [86]. For example, a prolonged stance or double support phases, a reduction in gait velocity, and a reduction in step length, are all relevant parameters for diagnosing pathological gaits and assessing functional outcomes after rehabilitation.

These investigations will be part of the future improvement of this study. The current preliminary results encourage us to pursue this line of research to achieve a solution based on a single RGB-D sensor for the analysis and reliable assessment of the gait, which is suitable for home monitoring and rehabilitation. For this purpose, the system will be equipped with a dedicated easy-to-use user interface, based on body movements, to ensure the system can be easily self-managed, even by people with poor skills in using technology. This latter point is fundamental for deploying the system in unsupervised environments (e.g., patients’ homes). Future home experimental campaigns will also include the evaluation of the system’s usability and acceptability through the administration of dedicated questionnaires, to collect positive and negative feedback from the participants to further improve the solution.

### Limitation

Although the Microsoft Kinect© v2 was discontinued several years ago, it is still widely used for clinical research. However, our study does not rely on the particular RGB-D sensor employed. The commercial availability of other RGB-D sensors and body tracking libraries will allow the current analysis to be improved and results to be obtained using non-invasive technologies. For example, recent studies [87,88] have shown the higher performance and accuracy of Microsoft Kinect© Azure, which is the new RGB-D sensor that replaced the Microsoft Kinect© v2. These data make us confident that we will be able to replicate the algorithms and improve the preliminary results in our subsequent studies.

## 5. Conclusions

This paper presents a solution for estimating gait patterns and parameters of post-stroke individuals using a single camera approach, based on a 3D RGB-D sensor and a short walkway. This solution is suitable for monitoring improvement or worsening of gait disorders in domestic, constrained, or unsupervised environments, where gold standard systems are unsuitable. For this purpose, a cohort of post-stroke individuals was assessed for gait impairments. To verify the accuracy, robustness, and reliability of the measurements, a subset of the traditional spatiotemporal parameters estimated by the proposed system was compared with that estimated by a simultaneous instrumented 3D gait analysis using an optoelectronic system. In addition, parameters related to movements in the body center of mass were also included in this study as features that characterize the gait patterns. The analysis was performed by defining a virtual gait analysis path compatible with the typical space constraints of domestic environments. Preliminary results highlighted the good agreement, accuracy, and correlation between the gait parameters estimated by the two systems, with the exception of step width, in agreement with other studies. In addition, the most highly correlated parameters also showed a strong correlation with the Timed Up and Go test, which is one of the most common tests used to evaluate gait impairments in clinical practice. The preliminary results also suggest that the proposed solution can be employed as an intermediate tool for gait analysis, through a subset of the traditional spatiotemporal parameters, to objectively monitor changes in gait on a shorter walking path. The graphical representation of the most relevant parameters using radar charts provides an immediate indication of the patient’s gait pattern and facilitates the comparison of gait performance over time. The availability of a low-cost and non-invasive tool is essential for monitoring gait strategies, especially in the home setting. This allows both the exploration of the potential of gait analysis in environments where the traditional 3D-GA is not usable, and its combination with remote rehabilitation treatments to address the training and recovery of this motor function for people’s daily safety and independence.

## Figures and Tables

**Table 1:** Spatiotemporal and COM parameters with meaning and estimation for the 3D-GA and RGB-D systems.

**Spatiotemporal** **Parameters [Unit]**	**Meaning**	**3D-GA** **System**	**RGB-D** **System**
Step length [m]	Length of step	Distance between the point of initial contact of one foot and the point of initial contact of the opposite foot	Distance in “depth” between the start of the “stationary” period of one ankle and the start of the “stationary” period of the opposite
Stance duration [%]	Foot support phase	Duration from heel strike to toe off of the same foot, as percentage of gait cycle	Duration of the “stationary” condition of one ankle, as the percentage of gait cycle
Double support duration [s]	Support on both feet	Duration of the support phase on both feet	Duration of the “stationary” phase for both ankles
Mean velocity [m/s]	Average gait speed	Instantaneous speed as the ratio between step length and step time	Instantaneous speed as the ratio between step length and step time
Cadence [step/min]	Rate	Number of steps per minute	Number of steps per minute
Step width [m]	Step width	Distance between line of progression of one foot and the line of progression of the other	Distance between the line of progression of one ankle and the line of progression of the other
**Center of Mass** **Parameters [Unit]**	**Meaning**	**3D-GA** **System**	**RGB-D** **System**
ML sway [m]	Medio-Lateral excursion	Peak-to-peak COM_BODY_ sway (ML direction)	Peak-to-peak COM_HIP_ sway (ML direction)
V sway [m]	Vertical excursion	Peak-to-peak COM_BODY_ sway (V direction)	Peak-to-peak COM_HIP_ sway (V direction)

**Table 2:** Results of the statistical analysis on the median and first quartile values for spatiotemporal and COM parameters estimated for the two systems. Only step width shows a significant difference.

**Spatiotemporal** **Parameters [Unit]**	**3D-GA ** **System**	**RGB-D ** **System**	***p*** **-Values (Effect Size)**
Step length [m]	0.490 (0.217)	0.464 (0.170)	0.481 (0.145)
Stance duration [%]	65.000 (6.970)	67.500 (9.000)	0.405 (0.180)
Double support duration [s]	0.415 (0.245)	0.510 (0.387)	0.565 (0.123)
Mean velocity [m/s]	0.79 (0.412)	0.80 (0.404)	0.991 (0.002)
Cadence [step/min]	93.900 (24.200)	95.240 (26.590)	0.972 (0.007)
Step width [m]	0.225 (0.053)	0.194 (0.063)	0.002 * (0.666)
**Center of Mass** **Parameters [Unit]**	**3D-GA** **System**	**RGB-D** **System**	***p*** **-Values (Effect Size)**
ML sway [m]	0.105 (0.058)	0.092 (0.045)	0.555 (0.269)
V sway [m]	0.041 (0.013)	0.051 (0.050)	0.069 (0.689)

* *p* < 0.05.

**Table 3:** Correlation between spatiotemporal and COM parameters between 3D-GA and RGB-D systems: results for Spearman’s correlation coefficient, intra-class correlation (ICC) and accuracy.

**Spatiotemporal** **Parameters**	**Spearman’s Correlation**	**ICC**	**ACC_%_**
Step length	0.77 *	0.86	9.88%
Stance duration	0.72 *	0.73	5.52%
Double support duration	0.91 *	0.94	18.51%
Mean velocity	0.90 *	0.94	1.47%
Cadence	0.71 *	0.94	8.61%
Step width	0.34	0.47	22.22%
**Center of Mass** **Parameters**	**Spearman’s Correlation**	**ICC**	**ACC_%_**
ML sway	0.81 *	0.89	3.39%
V sway	0.70 *	0.72	16.08%

* *p* < 0.05.

**Table 4:** Spatiotemporal parameters for the paretic and non-paretic sides of gait patterns shown in Figure 7.

	**#1—Figure 7** **a**		**#2—Figure 7** **b**	
**Spatiotemporal** **Parameters**	**Paretic Side**	**Non-Paretic Side**	**Paretic Side**	**Non-Paretic Side**
Step length [m]	0.50	0.56	0.15	0.23
Stance duration [%]	62.10	63.24	86.32	84.76
Double support duration [s]	0.31	0.34	1.86	1.85
Mean velocity [m/s]	0.87	0.94	0.14	0.15
Step width	0.20	0.16	0.16	0.16

**Table 5:** Correlation between gait parameters and TUG test: coefficients and *p*-values of Spearman’s correlation for 3D-GA and RGB-D systems.

**Spatiotemporal** **Parameters**	**3D-GA** **System**	**RGB-D** **System**
Step length	−0.86 (1.50 × 10^−3^)	−0.88 (8.40 × 10^−4^)
Stance duration	0.93 (2.59 × 10^−4^)	0.95 (2.59 × 10^−5^)
Double support duration	0.96 (6.70 × 10^−6^)	0.96 (1.50 × 10^−5^)
Mean velocity	−0.89 (6.07 × 10^−4^)	−0.92 (2.00 × 10^−4^)
Cadence	−0.88 (8.63 × 10^−4^)	−0.87 (1.10 × 10^−4^)
Step width	−0.06 (0.82)	0.14 (0.76)
**Center of Mass** **Parameters**	**3D-GA** **System**	**RGB-D** **System**
ML sway	0.51 (0.13)	0.30 (0.38)
V sway	−0.42 (0.23)	−0.38 (0.28)
